# The ability of *Arabidopsis* to recover from Basta and its application in isolating *Cas9*-free mutants

**DOI:** 10.3389/fpls.2024.1408230

**Published:** 2024-10-16

**Authors:** Shahbaz Ahmed, Anna K. Hulbert, Xin Xin, Michael M. Neff

**Affiliations:** ^1^ The Department of Crop and Soil Sciences, Washington State University, Pullman, WA, United States; ^2^ Graduate Program in Molecular Plant Sciences, Washington State University, Pullman, WA, United States

**Keywords:** CRISPR-Cas9, gene editing, Cas9 T-DNA, mutants, *Arabidopsis thaliana*, Basta selection

## Abstract

After successfully performing *Agrobacterium-*mediated CRISPR-Cas9-based gene editing in plants, isolation of the *Cas9* T-DNA is essential for the stable inheritance of induced mutations. Here, we report a simple technique that allows the isolation of *Cas9*-free mutants, eliminating the need for outcrossing or other intricate methods. This method is based on the ability of Basta-sensitive *Arabidopsis thaliana* seedlings, which generally perish, to recover and grow once transplanted to Basta-free growth media. By growing gene-edited heterozygous populations of single-locus insertion Basta-resistant plants on Basta selection media, plants lacking the *Cas9* T-DNA can be identified. These pale-looking plants lacking Cas9 are then rescued on media lacking the Basta to recover *Cas9*-free plants. The ability of seedlings to recover from Basta selection was also studied in camelina, canola, and wheat. All three crops showed different recovery rates, with wheat demonstrating the highest recovery once transplanted from Basta to normal growth media. In summary, our findings demonstrate that by harnessing the recovery capability of Basta-sensitive seedlings, we can effectively identify and rescue plants lacking the Cas9 T-DNA, enabling the isolation of Cas9-free mutants in *Arabidopsis* and potentially extending to other crops.

## 1 Introduction

Breeders have contributed to altering animal and plant genomes for years through traditional hybridization and selection to produce new genetic combinations for improved commodity development. This process, however, can take years (Orton, 2020). To overcome this problem, engineered nucleases such as zinc finger nucleases (ZFNs) and transcription activator-like effector nucleases (TALENs) were discovered to effectively carry out specific genetic changes in plant genomes (Carroll, 2011; Nemudryi et al., 2014). Despite their ability to carry out site-specific gene editing, engineering through ZFNs and TALENs is time-, effort-, and cost-consuming. Clustered regularly interspaced short palindromic repeats (CRISPR) and CRISPR-associated nucleases such as Cas9 have emerged recently as breakthrough tools for genome editing studies (Doudna and Charpentier, 2014).

First reported in 2011, these molecular scissors are now widely used in labs to make precise cuts in genomic DNA (Zhu et al., 2020). CRISPR was first discovered in the genome of *Escherichia coli* in 1987, where the interspaced sequences in the CRISPR are derived from past genome invasions (Ishino et al., 1987). There are three main participants in the CRISPR-Cas9 machinery: CRISPR-RNA (crRNA), trans-activating crRNA (tracrRNA), and a Cas nuclease protein. The transcription of the CRISPR loci, along with the tracrRNA(s) and Cas nuclease protein, acts as an adaptive bacterial defense against viral attacks. As its name states, the CRISPR locus has multiple palindromic repeats with spacer DNA between the repeats (Jiang and Doudna, 2017). To facilitate the cloning procedure and improve genome editing efficiency, scientists have engineered dual tracrRNA:crRNA as a single guide RNA (sgRNA) where a 20-nucleotide single-target matching sequence makes up the 5’ end and a secondary structure at the 3’end helps for Cas9 binding (Doudna and Charpentier, 2014). The double-stranded DNA break generated by CRISPR-Cas9 triggers the DNA repair mechanism. DNA repair by NHEJ can result in errors in the target gene, often resulting in deletions or insertions of bases, which can cause a coding frameshift, rendering the gene inactive (Jeggo, 1998). This technique is widely used today by laboratories in studying gene knockout phenotypes in a wide range of organisms. Plants are routinely edited for genes of interest in order to study knockout phenotypes without substantial off-target mutations, which can be associated with random mutagenesis approaches such as TILLING (Kurowska et al., 2011; Zaidi et al., 2020).

Despite the ability of CRISPR-Cas9 to efficiently induce desirable mutations, as long as the target sequence is present, the existing CRISPR-Cas9 complex can still induce additional mutations. This includes the possibility of additional insertions or deletions that could potentially revert the initial lesion back to a (near)-wild-type state. Thus, it is important to remove the CRISPR- Cas9 complex-containing T-DNA from the plants in order to ensure stable inheritance of the desired induced mutation (Gao et al., 2016). In addition, the prolonged presence of the CRISPR- Cas9 complex can cause potential off-target mutagenic activity, resulting in undesirable or unknown genomic mutations. The presence of multiple orthologs of a gene due to genome duplication can also increase the risk of off-target gene editing by the continued presence of the CRISPR- Cas9 complex (Zhang et al., 2018; Hahn and Nekrasov, 2019).

Removing the CRISPR-Cas9 complex from plant genomes can be achieved through several methods (Gao et al., 2016; He and Zhao, 2019; Kang et al., 2023; Yang et al., 2023). However, some of these techniques face challenges regarding their acceptability across different plant types. For example, the use of visual selectable markers such as the fluorescent protein DsRed to identify Cas9-transgene-free individuals may be difficult due to autofluorescence or in plants harboring a previously introduced fluorescent marker. In addition, the color or translucency of the seed coat may block the visualization of a fluorescent marker. A recently developed approach is to use grafting where the CRISPR- Cas9 complex can be transported from a transgenic rootstock to wild-type scions (Yang et al., 2023). Although this technique can result in transgene-free gene-edited seeds, it can only be reliably used in dicotyledonous plants.

Here, we report a novel but simple strategy to obtain *Cas9*-free mutants with a stable inheritance in *Arabidopsis thaliana* (*Arabidopsis*). In this approach, we used a Cas9+gRNA in a vector with a *phosphinothricin acetyltransferase gene (blpR)*, conferring resistance to Basta^®^ (glufosinate ammonium). Our approach to removing the CRISPR-Cas9 machinery from successfully gene-edited *Arabidopsis* plants is based on rescuing non-transgenic *Arabidopsis* seedlings from Basta-selection media. As a proof of concept, we targeted sites in the *At-Hook containing nuclear Localized 26* (*AHL26*) gene using a CRISPR-Cas9 complex. *AHL26* is part of the *AHL* gene family, which features a conserved AT-hook motif at their N-terminal end and a plant and prokaryote conserved (PPC) domain, also known as the domain of unknown function #296 (DUF296), at their C-terminal end. Our lab routinely works with this family of genes to study seedling development and flowering time (Street et al., 2008; Zhao et al., 2013, 2014; Favero et al., 2016; Tayengwa et al., 2020; Jacques et al., 2022). After obtaining CRISPR-generated *AHL26* mutants, we grew the segregating generation of *Cas9* mutants on Basta selection and rescued and sequenced the non-transgenic offspring lacking the *Cas9* construct. Once identified, we screened *Cas9*-free mutants for two more generations to demonstrate the heritability of the mutation. Using this strategy, we were able to isolate stable *Cas9*-free mutants in *Arabidopsis* for the *AHL26* gene. We also demonstrate how the principles of this technique can be applied to field crops such as canola and wheat.

## 2 Material and methods

### 2.1 Growth media conditions

The growth media used for these studies are described in (Jacques et al., 2020). Briefly, selection media contained 0.5× Linsmaier and Skoog (LS) modified basal medium, 1.5% (w/v) sucrose, and 0.8% (w/v) Phytoblend as a solidifying agent (Caisson, Smithfield, UT) with appropriate antibiotics. Gellan gum (PhytoTechnology Laboratories, Inc.) was used as a solidifying agent in non-selection media to grow rescued seedlings along with 0.5× Linsmaier and Skoog (LS) modified basal medium and 1.5% (w/v) sucrose to ensure optimum plant growth. Gellan gum was used for seedling development assays based on the observations that this gelling agent is useful for discerning subtle seedling phenotypes on non-selection media (Jacques et al., 2020).

### 2.2 Selection of transformants

All *Arabidopsis thaliana* plants used in this study are in the Columbia (Col-0) background. CRISPR-*Cas9* transgenics were obtained through the floral-dip method (Clough and Bent, 1998). The putative primary (T1) transgenic seeds were grown on selection media containing 25mg/L Basta (Oakwood Chemicals, Estill SC). Thirty plants of 20 independent transformants in the T2 generation were screened on selection media to identify lines containing single locus insertion based on chi-square analysis for a predicted 3^resistant^:1^sensitive^ segregation ratio. A chi-square test was conducted on individual lines to determine if the P-value exceeded the significance threshold of 0.05. Lines with a P-value greater than 0.05 were considered to have a single locus insertion. This confirmation was based on the T2 generation and not repeated in later generations. Multiple transgenic T2 lines, heterozygous for the *Cas9* T-DNA, with single-locus insertions were used to obtain T-DNA-free CRISPR-*Cas9* mutants.

### 2.3 Plasmid and constructs

The *AHL26*-specific guide RNA (gRNA) was designed using the E-CRISPR design tool (http://www.e-crisp.org/E-CRISP/). The resulting top and bottom gRNAs (**Supplementary Figure S1**) included 5’-GGTCA-3’ and 5’-AAAC-3’ cohesive ends to assist in vector annealing. Annealed and phosphorylized oligos were ligated in the pYPQ131 gRNA expression vector (Lowder et al., 2015). This construct and the Cas-9 containing pYPQ154 (Lowder et al., 2015) were cloned into pED15 using MultiSite Gateway™ Technology. pED15 contains a gene for Basta-based transgenic selection. The modified pED15 with Cas-9 and gRNA targeting *AHL26* was named pAK4 and the complete sequence of the vector is available in **Supplementary Figure S2**. Sequence analysis was performed at every step to confirm the successful ligation of the guide RNAs. The final construct was then used to obtain two independent transgenic free mutant lines (*ahl26*-*1*, *ahl26-2*).

### 2.4 Sequencing for mutant selection

DNA from the 50 plants from 5 independent single locus insertion lines was extracted using a DNeasy Plant Mini Kit (Qiagen, Valencia, CA) following the manufacturer’s protocol. The PCR was performed using forward and reverse primers spanning the *AHL26* gene (S1). The PCR product was cleaned, and potential mutants were sequenced using the Sanger sequencing method. The sequence from potential mutants was aligned to wildtype *AHL26* to identify CRISPR-Cas9-generated mutations.

### 2.5 Rescuing Basta-sensitive seedlings

Twenty transgenic *Arabidopsis* seeds, replicated three times for each independent single locus insertion line, heterozygous for *phosphinothricin acetyltransferase gene (blpR)* conferring resistance to Basta were sterilized and grown on selection media containing 25mg/L Basta for 8-days (Tayengwa et al., 2020). The segregating sensitive non-transgenic seedlings (**Supplementary Figure S3**) were then transferred using forceps to sterile media without a selection agent. Seedlings were classified as sensitive if they exhibited pale leaves, failed to accumulate chlorophyll, conferred closed cotyledons, had stunted growth, or failed to develop leaves when grown on the media (Harrison et al., 2006). Seedlings were kept on the non-selection plates until leaves turned green before being transferred to soil and grown in standard greenhouse conditions. Normal growth practices were applied for the rest of the plant growth cycle. The seedlings were categorized as unable to recover based on specific criteria, including the presence of pale leaves, lack of new leaf development, and persistent stunted growth after being transferred from selective media to normal growth conditions. Seedlings that exhibited these symptoms without showing any signs of improvement or growth were deemed unable to recover.

Basta, kanamycin, and hygromycin are the three widely used markers in transgenic selection studies. To understand if the *Arabidopsis* seedling recovery can also occur in kanamycin and hygromycin, twenty wild-type *Arabidopsis* seeds replicated thrice were grown on media containing 50mg/L kanamycin or 20mg/L hygromycin. Transgenic *Arabidopsis* seedlings, resistant to kanamycin and hygromycin, were grown on respective selection media as control checks to compare the growth of sensitive versus resistant seedlings. These specific concentrations were selected based on published studies that identify them as effective for distinguishing transgenic from non-transgenic seedlings (Harrison et al., 2006; Ee et al., 2014). Discolored seedlings after 8-days from both selection agents were moved to normal growth media to observe their recovery and growth. Sensitive seedlings were deemed ready for recovery when they exhibited clear stress symptoms, such as pale leaves and stunted growth, making them easily distinguishable from the control seedlings.

The recovery of plants was quantified as a percentage. Twenty *Arabidopsis* plants replicated in triplicates were initially grown on either Basta, kanamycin and hygromycin media and, after eight days, were transferred to normal growth media. Plant recovery after 15 days was recorded as qualitative data, categorized as either “recovered” or “not recovered”. The number of recovered plants was then counted and divided by the total number of plants to calculate the recovery percentage.

Additionally, fifteen wild-type seeds each from camelina, canola, and wheat, replicated in triplicate, were surface sterilized and grown on media supplemented with 25 mg/L Basta (Cardoza and Stewart, 2004). Discolored seedlings from all three crops were transferred to non-selection media to initiate growth recovery. The Basta-affected seedlings from canola and wheat were also transplanted directly into the soil to observe growth recovery. The criteria for recovery of wheat, camelina and canola seedlings after being transferred from selection to non-selection media included the observation of healthy, green leaves, initiation of new leaf growth, and a visible increase in overall plant vigor within 15 days after transfer. Seedlings that demonstrated these signs of recovery, without showing symptoms of necrosis or stunted growth, were considered to have successfully recovered.

## 3 Results

### 3.1 Rescue of Basta-sensitive *Arabidopsis* seedlings

**Figure 1:**
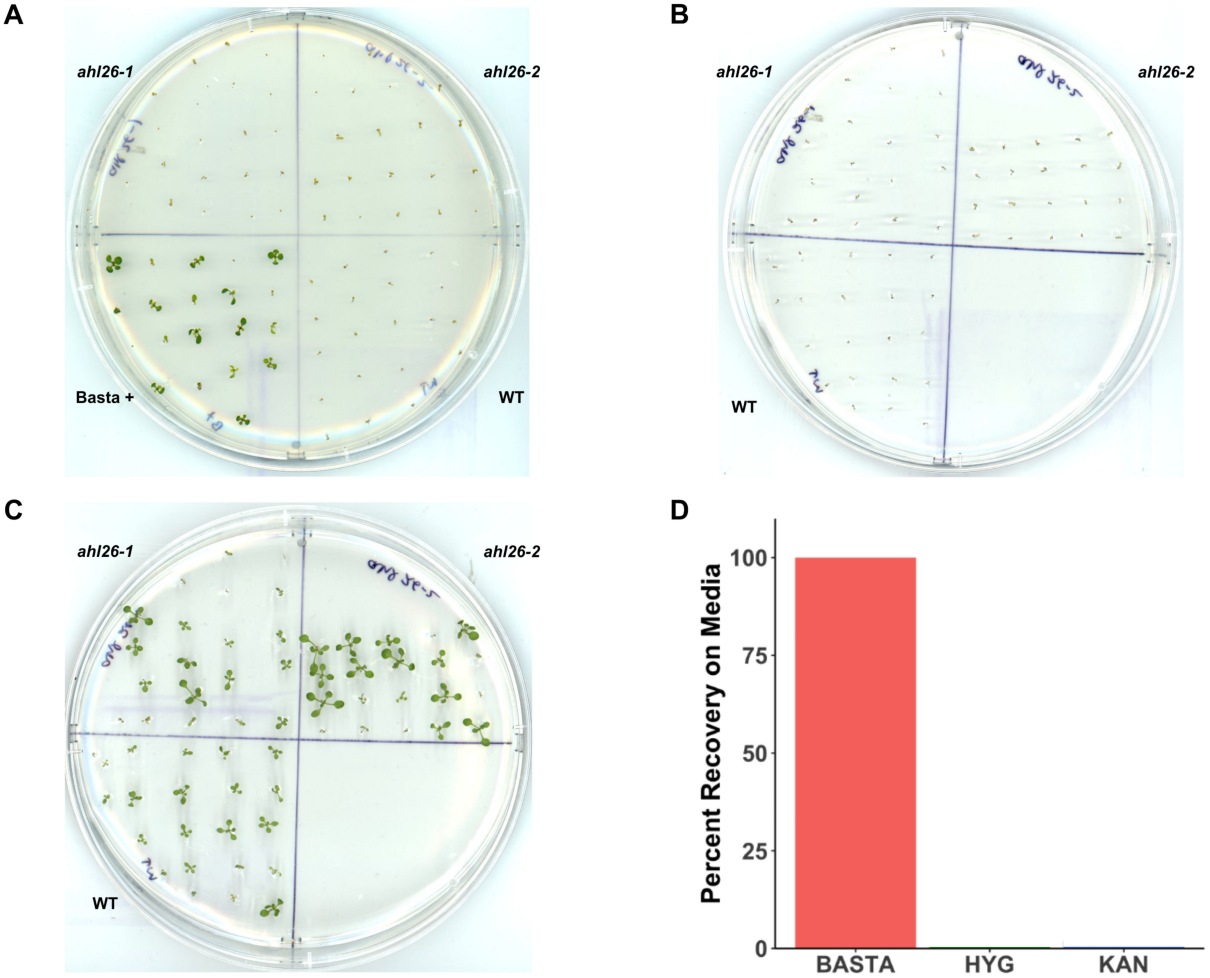
Recovery of *Arabidopsis* seedlings from selection media. **(A)**. *Arabidopsis* seedlings growing on Basta 25mg/L. The plate is divided into four parts. Col-0, Basta^+^ (a positive check for Basta media), ahl26-1, and ahl26-2 lacking Cas9-T-DNA. **(B)**. The sensitive seedlings from the Basta plate are transferred to non-selection media after the appearance of symptoms. **(C)**. The transplanted seedlings from **(B)** plates after recovery. **(D)**. The percentage of recovery from Basta, Hyg, and Kan plates when transferred to non-selection media after the appearance of symptoms. n = 20, replicates = 3. Error bars are absent because all replicates showed either 100% recovery or 100% mortality for the Basta, kanamycin, and hygromycin treatments.

To examine the ability of non-transgenic *Arabidopsis* to recover from the selection of the herbicide Basta, seedlings were first grown on Basta selection media as described in the Methods. The 8-days old pale-appearing selection-sensitive seedlings from Col-0 (S3) and two independent transgenic free mutant lines (*ahl26*-*1*, *ahl26-2*) were then transferred to media without Basta to begin seedling recovery. Using this method, we were able to rescue and recover 100 percent of the discolored non-transgenic seedlings from the previous Basta selection (**Figures 1A-C**). All the recovered seedlings were then grown to seed-set in the greenhouse after being transplanted to soil. While growing on Basta media, differences in size were observed among individuals, potentially influenced by factors such as placement on the media, seed size variation, and other unidentified mechanisms. Subsequently, during recovery on Basta-free media, variations in recovery rates were noted, potentially stemming from initial size differences, thus contributing to the observed variations in size among the seedlings. Despite the different growth rates at media, all the recovered seedlings exhibited normal growth patterns throughout their life cycle.

Kanamycin and hygromycin along with Basta are the three widely used transgenic selection agents. Unlike Basta, which is an herbicide, kanamycin and hygromycin are antibiotics. To examine the potential application of the Basta seedling rescue method with other selection agents, we studied the ability of *Arabidopsis* to recover from kanamycin or hygromycin selection. Unlike Basta, none of the seedlings grown on kanamycin or hygromycin selection media were able to recover and grow to adulthood (**Figure 1D**). These findings indicate that our method for *Cas9* isolation is effective solely with the Basta selection marker.

### 3.2 Selection of CRISPR-*Cas9*-free mutants

**Figure 2:**
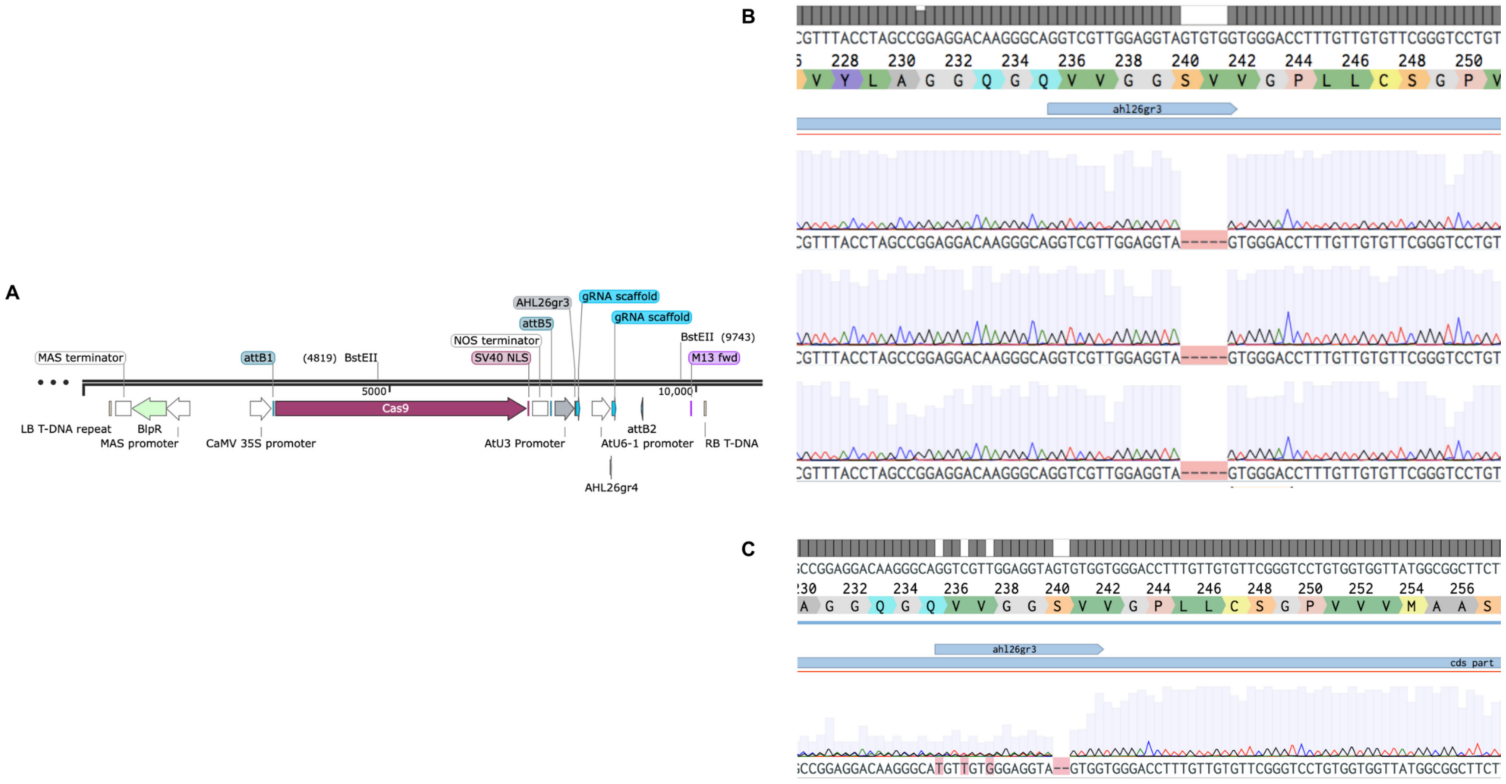
CRISPR-Cas9 to carry out mutations in AHL26. **(A)**. Plasmid with gRNA targeting the AHL26 exon with the Basta gene for transgenic selection. **(B)**. The heritable five-base deletion in ahl26-1 was confirmed through PCR-based sequencing in non-transgenic Cas9-free Arabidopsis plants. **(C)**. The heritable two-base deletion in ahl26-2.

**Figure 3:**
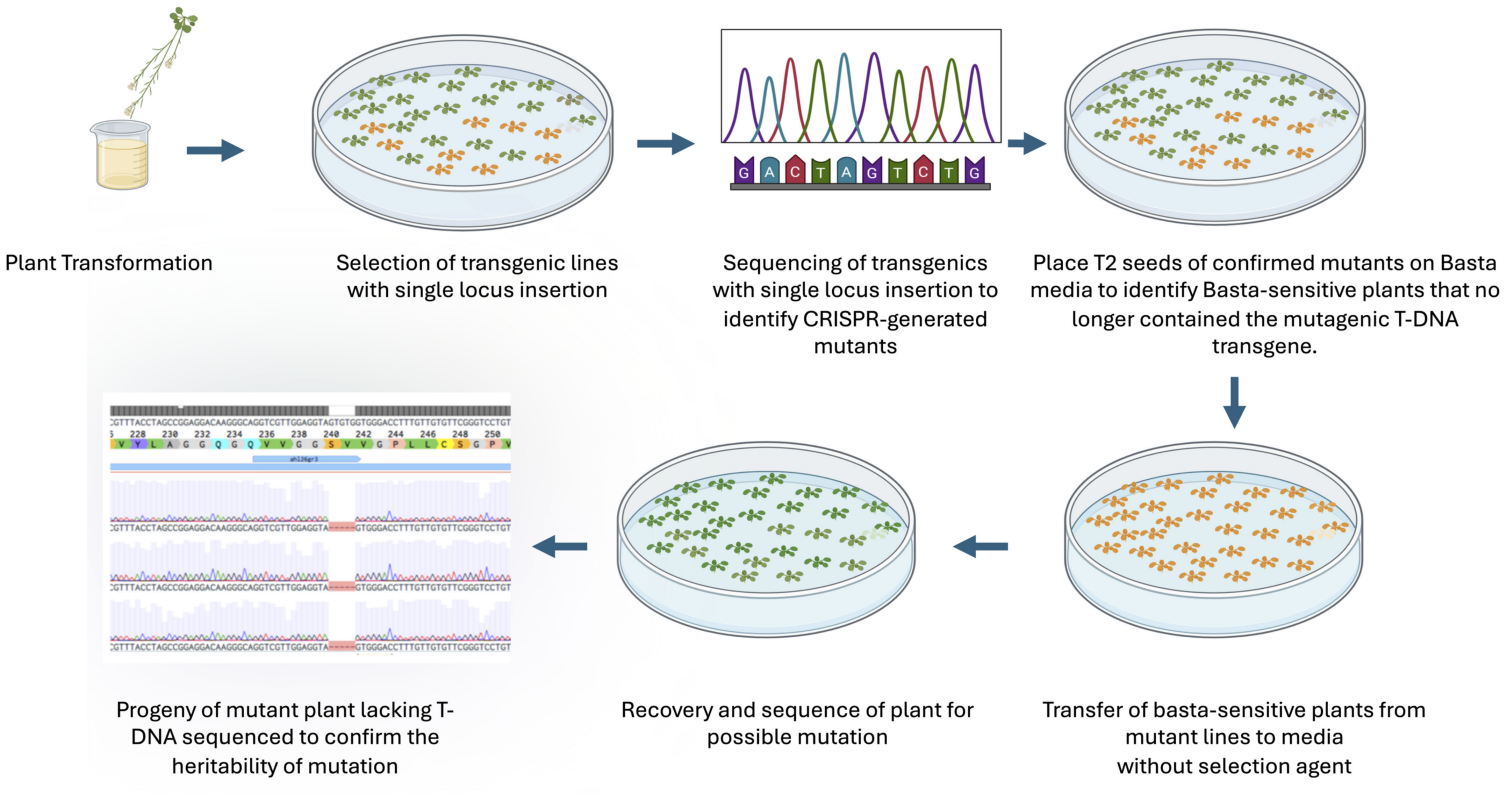
The flow diagram of isolation of Cas9-free mutants. The figure illustrates the process of obtaining heritable CRISPR-induced Cas9-free mutations. Key steps include selecting single locus insertions, sequencing to identify mutations, and using Basta sensitivity to isolate transgene-free plants, followed by confirming mutation inheritance in subsequent generations.

To test whether Basta-recovered seedlings can be used to identify *Cas9*-free mutants, we designed guide RNAs to mutate the *AHL26* gene in *Arabidopsis thaliana*. The *AHL26* gene was amplified by PCR from T2 plants derived from single-locus transgene insertion events. These PCR products were then sequenced to identify two *AHL26* mutants, *ahl26-1* and *ahl26-2* harboring five- and two-base deletions respectively (**Figure 2**). Seeds of the CRISPR-generated mutant plants heterozygous for *Cas9* were then grown on Basta selection media to identify the Basta-sensitive plants lacking the *Cas9* construct. In the next step, the non-transgenic *Arabidopsis* plants sensitive to Basta media were transferred to media without any selection agent. The sequencing of recovered non-transgenic plants showed similar five- and two-base deletions at the target site, resulting in the successful isolation of *Cas9*-free mutants. In the next step, *Cas9*-free mutants were grown and sequenced for *AHL26* target mutation for two more generations to study the inheritance of the mutation. The workflow for this process is presented in **Figure 3**.

### 3.3 Recovering seedlings of wheat, canola, and camelina

**Figure 4:**
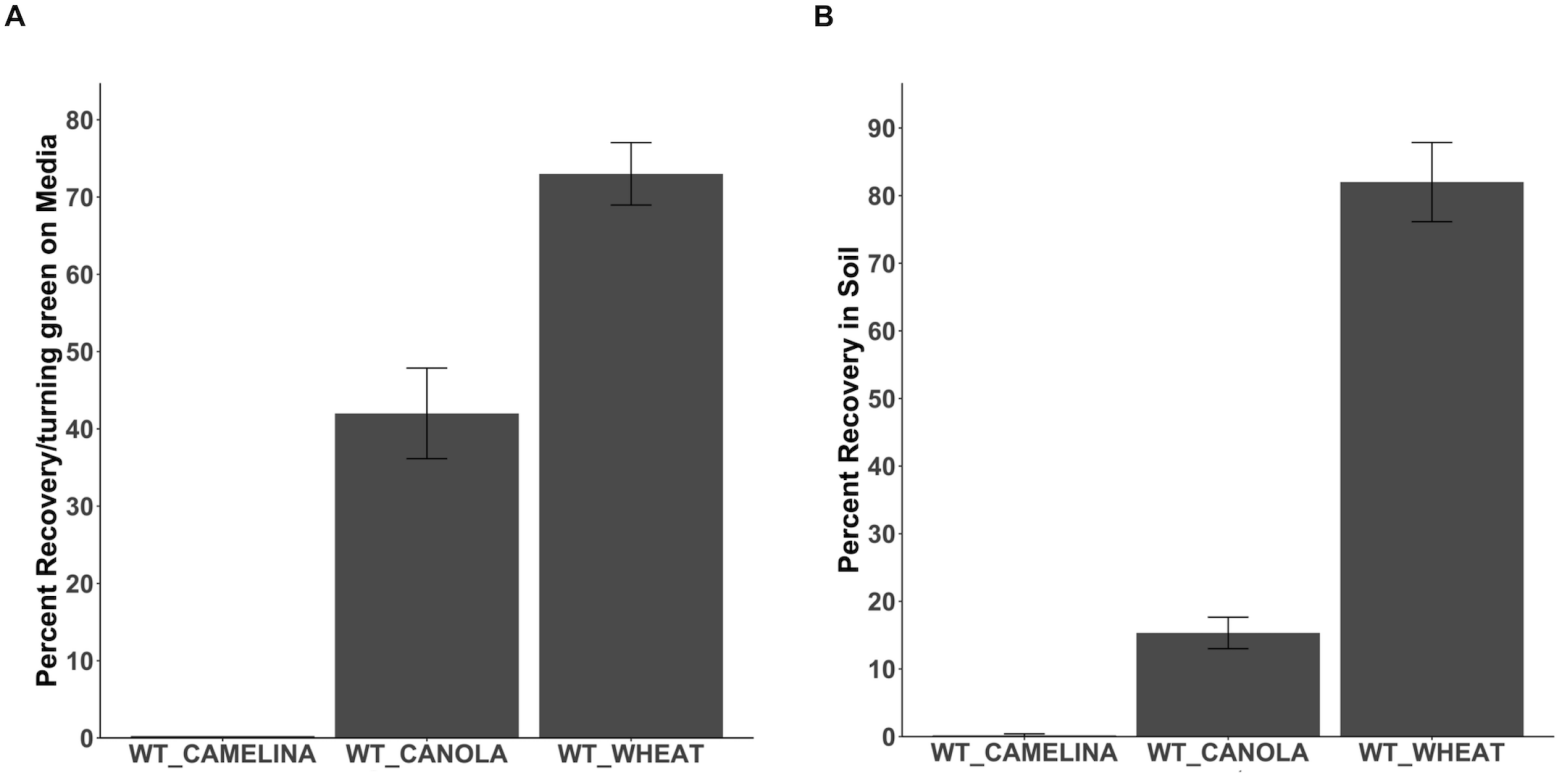
Recovering Wheat, Camelina, and Canola seedlings from Basta. **(A)**. The percentage recovery/turning green in camelina, canola, and wheat at non-selection media after initial growth on Basta media **(B)**. The percentage recovery and growth of camelina, canola, and wheat after transferring from selection media directly to the soil. N= 15, replication=3. The error bar donates the SEM.

To study the ability of other non-transgenic crops to recover from Basta selection, we grew wild-type wheat, canola, and camelina on selection media containing 25mg/L of Basta. Chlorotic seedlings were transferred to non-selection media to observe growth recovery. No recovery in camelina seedlings was observed when transferred from selection to non-selection media (**Figure 4A**). (**Figure 4A**; **Supplementary Figure S4**). Canola seedlings showed less than 20 percent recovery in the non-selection media (**Figure 4A**). Direct transfer of canola seedlings from Basta selection media to the soil showed similar results (15 percent) (**Figure 4B**; **Supplementary Figure S5**). Over 90 percent of wheat seedlings were recovered when transferred to non-selection media (**Figure 4A**). Wheat showed similar results when seedlings were directly transferred to soil in greenhouse conditions (82 percent) (**Figure 4B**; **Supplementary Figure S6**). According to these findings, Basta was lethal to camelina, while canola and wheat exhibited recovery following exposure.

## 4 Discussion

In this study, we report a technique to obtain *Cas9*-transgene-free *Arabidopsis* mutants and its potential application in other crops such as wheat and canola. This method is based on the ability of some plants to recover from Basta selection. Basta herbicide has an active ingredient, glufosinate-ammonium (Hoerlein, 1994; Takano and Dayan, 2020) with a mode of action that inhibits the glutamine synthetase enzyme. Glutamine synthetase plays a role in nitrogen metabolism in plants, and the inactivation of this enzyme results in the disruption of multiple metabolic pathways, including photosynthesis (Krogmann et al., 1959; Wendler et al., 1990; Wild and Wendler, 1993; Dan Hess, 2000; Dayan et al., 2019). The inhibition of glutamine synthetase results in the accumulation of toxic amounts of ammonia in the chloroplast (Bernard and Habash, 2009; Takano and Dayan, 2020). Plants exposed to this herbicide stop growing, resulting in death approximately two weeks later. Our lab routinely uses the *phosphinothricin acetyltransferase* gene *(blpR)* as a positive selection marker in plasmids for selecting transgenic plants which have been exposed to Basta. The *blpR* gene in transgenic plants confers resistance to glufosinate-ammonium by acetylating it to create an inactive conjugate, which allows plants to grow on growth media with the Basta selection agent (Block et al., 1987).

During our studies with transgenics plants expressing the *blpR* gene, we observed the ability of the non-transgenic plants to recover their growth once the plants were moved to media without the Basta selection agent. Since Basta doesn’t directly disrupt the photosynthesis system, the absence of the herbicide allowed some plants to regain their normal growth. Non-transgenic *Arabidopsis* seedlings growing on Basta media display stunted growth with discolored leaves. We observed 100 percent growth recovery once the affected seedlings were moved to normal growth media (**Figure 1D**). This method is consistent with findings in a field where injury to plants due to herbicide application can be corrected by removing the herbicide contaminated soil with clean soil (Mathers, 2006). The recovered seedlings were then able to grow normally in soil under greenhouse conditions. In contrast, *Arabidopsis* seedlings grown on kanamycin or hygromycin selection media could not recover their growth after being transferred to non-selection growth media (**Figure 1D**). Basta, being an herbicide, differs from kanamycin and hygromycin in the mode of action as both antibiotics interfere with protein synthesis in the chloroplast, causing permanent damage to plants (Davies et al., 1965; Weisblum and Davies, 1968; Davis, 1987; Hoerr et al., 2016). It is important to clarify that the purpose of our study is not to compare the effectiveness of different selection agents against each other. Instead, we aimed to determine which selection agents allow seedlings to be successfully rescued after selection. By testing Basta, kanamycin, and hygromycin, we sought to identify whether our seedling rescue method could be applied across different selection agents. Our findings show that this recovery phenomenon is specific to Basta, suggesting that the method is most effective when using Basta as the selection marker.

Non-transgenic siblings are plants from the same genetic background as transgenic plants but do not contain transgene of interest. These plants can be obtained by growing the progeny of a heterozygous transgenic plant on selection media to identify sensitive non-transgenic siblings. These siblings can serve as a control group, allowing for more accurate comparisons by minimizing genetic variation unrelated to the transgene. The recovery of Basta-sensitive seedlings from Basta-selection media provides an opportunity to easily identify non-transgenic siblings in plant transformation studies. The isolation of non-transgenic siblings not only allows to select *Cas9*-transgene-free mutants in *Arabidopsis*, as reported in this study, it also offers researchers several advantages in experimental design and result interpretation. For example, comparing transgenic plants to closely related non-transgenic siblings is particularly important when studying traits that may be influenced by minor genetic variations caused by the transformation process. Another more labor-intensive approach for identifying non-transgenic siblings in plant transformation studies involves self-pollination of heterozygous transgenic plants or backcrossing to the wild type, which is then followed by PCR assays or transgene selection analysis in paired sibling populations.

While other methods isolating *Cas9*-free mutants have been described, such as fluorescent-based isolation or grafting, these systems may not be compatible with all plant species or varieties (Gao et al., 2016; Yang et al., 2023). For example, although DsRed fluorescence selection offers a simple alternative for selecting Cas9-free plants, its application is limited to seeds with translucent seed coats, such as *Arabidopsis* and camelina. Plant seeds with thick seed coats, such as canola and wheat, limit the detection of DsRed fluorescent signals. In addition, grafting presents a practical challenge with the need for special equipment along with skilled personnel. It also has a potential limitation in monocots due to the lack of a cambium layer of cells. The ease of identifying and isolating non-transgenic plants using our method from the progeny of CRISPR-Ca9 generated mutants offers a simple and reproducible alternative for isolating *Cas9*-free mutants.

Although our method offers a simple alternative to isolate Cas9-free *Arabidopsis* plants, the method only allows the isolation of T-DNAs that still contain a selection marker gene. It does not track unknown T-DNA pieces inserted into the plant genome, the isolation of which is necessary to make true non-transgenic plants. Thus, if true non-transgenic plants are the goal, a whole genome sequence will be necessary to determine the presence of any such foreign DNA elements in any of the methods used for transgenes selection such as DsRed. The efficiency of mutation is highly dependent on the promoter driving the CRISPR gene. In our study, the mutation efficiency of the CRISPR system used was relatively low. However, recent advancements in CRISPR systems, such as those described by (Tsutsui and Higashiyama, 2017), have achieved mutation efficiencies exceeding 90%. With these more efficient CRISPR systems, our method could be significantly more effective, increasing the likelihood that non-transgenic segregating plants from mutant lines would harbor the desired mutation.

In addition to *Arabidopsis*, we investigated the recovery of field crops like camelina, canola, and wheat from Basta selection media. Based on our defined recovery criteria, camelina failed to recover after being moved from selection to non-selection media. The lack of recovery in camelina from Basta media suggests that our method may be limited in effectiveness for crops other than *Arabidopsis*, canola, and wheat. Fortunately, DsRed system offers a reliable transgene selection method for camelina (Sharma Koirala and Neff, 2020) with proven efficacy in isolating Cas9-free mutants within this species (Gao et al., 2016). The response of non-transgenic canola to Basta selection involved growth slowdown followed by leaf yellowing, with varying recovery rates upon transplantation to normal growth media. Although canola seedlings had a lower recovery percentage in soil than *Arabidopsis*, more seedlings exhibited green hypocotyls, suggesting potential for tissue culture approaches. Conversely, wheat had a higher recovery percentage in both media and soil, most likely attributed to its tightly packed crown structure and higher regenerative ability compared to canola (Huang and Taylor, 1993; Shang et al., 2021). These findings underscore the versatility of our Basta recovery methods across different crops, each presenting unique challenges and advantages. For our camelina, canola, and wheat experiments, we did not include a Basta control due to the unavailability of transgenic lines for these plants. As a result, further testing is required to fully assess the effectiveness of the Basta recovery method in eliminating trans-mutagenic CRISPR-Cas9. However, we anticipate that the recovery observed in this study could be applicable to segregating transgenic plants as well.

## Data Availability

The raw data supporting the conclusions of this article will be made available by the authors, without undue reservation.
