# Supplementary material for: The ability of Arabidopsis to recover from Basta and its application in isolating Cas9-free mutants
Source: Front Plant Sci. 2024 Oct 16;15:1408230. doi: 10.3389/fpls.2024.1408230 (PMC11521829; doi:10.3389/fpls.2024.1408230)
Supplement: Supplementary file 1 [file DataSheet1.docx]

| Name | Sequence (5’-3’) | Description |
| --- | --- | --- |
| AHL26-gR3-top | ggtcaGGTCGTTGGAGGTAGTGTGG | CRISPR |
| AHL26-gR3-bottom | aaacCCACACTACCTCCAACGACCt | CRISPR |
| AHL 26-G-F1 | AATTCCAGCTTGATGTATT | to sequence ahl26 |
| AHL 26-G-R1 | GCCGTCTTCAACTTCACGTCAT | to sequence ahl26 |

**S1: Primers used in the study**

ATGTTGAATACTCATttattatttccttcctcttttctacagtatttaaagataccccaagaagctaattataacaagacgaactccaattcactgttccttgcattctaaaaccttaaataccagaaaacagctttttcaaagttgttttcaaagttggcgtataacatagtatcgacggagccgattttgaaaccgcggtgatcacaggcagcaacgctctgtcatcgttacaatcaacatgctaccctccgcgagatcatccgtgtttcaaacccggcagcttagttgccgttcttccgaatagcatcggtaacatgagcaaagtctgccgccttacaacggctctcccgctgacgccgtcccggactgatgggctgcctgtatcgagtggtgattttgtgccgagctgccggtcggggagctgttggctggctggtggcaggatatattgtggtgtaaacaaattgacgcttagacaacttaataacacattgcggacgtttttaatgtactgaattaacgccgaattaattcgagctcggatctGATAATTTATTTGAAAATTCATAAGAAAAGCAAACGTTACATGAATTGATGAAACAATACAAAGACAGATAAAGCCACGCACATTTAGGATATTGGCCGAGATTACTGAATATTGAGTAAGATCACGGAATTTCTGACAGGAGCATGTCTTCAATTCAGCCCAAATGGCAGTTGAAATACTCAAACCGCCCCATATGCAGGAGCGGATCATTCATTGTTTGTTTGGTTGCCTTTGCCAACATGGGAGTCCAAGATTCTGCAGtcaaatctcggtgacgggcaggaccggacggggcggtaccggcaggctgaagtccagctgccagaaacccacgtcatgccagttcccgtgcttgaagccggccgcccgcagcatgccgcggggggcatatccgagcgcctcgtgcatgcgcacgctcgggtcgttgggcagcccgatgacagcgaccacgctcttgaagccctgtgcctccagggacttcagcaggtgggtgtagagcgtggagcccagtcccgtccgctggtggcggggggagacgtacacggtcgactcggccgtccagtcgtaggcgttgcgtgccttccaggggcccgcgtaggcgatgccggcgacctcgccgtccacctcggcgacgagccagggatagcgctcccgcagacggacgaggtcgtccgtccactcctgcggttcctgcggctcggtacggaagttgaccgtgcttgtctcgatgtagtggttgacgatggtgcagaccgccggcatgtccgcctcggtggcacggcggatgtcggccgggcgtcgttctgggctcatCGATTCGATTTGGTGTATCGAGATTGGTTATGAAATTCAGATGCTAGTGTAATGTATTGGTAATTTGGGAAGATATAATAGGAAGCAAGGCTATTTATCCATTTCTGAAAAGGCGAAATGGCGTCACCGCGAGCGTCACGCGCATTCCGTTCTTGCTGTAAAGCGTTGTTTGGTACACTTTTGACTAGCGAGGCTTGGCGTGTCAGCGTATCTATTCAAAAGTCGTTAATGGCTGCGGATCAAGAAAAAGTTGGAATAGAAACAGAATACCCGCGAAATTCAGGCCCGGTTGCCATGTCCTACACGCCGAAATAAACGACCAAATTAGTAGAAAAATAAAAACTGACTCGGATACTTACGTCACGTCTTGCGCACTGATTTGAAAAATCTCAgAATTCCAATCCCACCAAAACCTGAACCTAGCAGTTCAGTTGCTCCTCTCAGAGACGAATCGGGTATTCAACACCCTCATACCAACTACTACGTCGTGTATAACGGACCTCATGCCGGTATATACGATGACTGGGGTTGTACAAAGGCAGCAACAAACGGTGTTCCCGGAGTTGCGCATAAGAAGTTTGCCACTATTACAGAGGCAAGAGCAGCAGCTGACGCGTATACAACAAGTCAGCAAACAGATAGGTTGAACTTCATCCCCAAAGGAGAAGCTCAACTCAAGCCCAAGAGCTTTGCGAAGGCCCTAACAAGCCCACCAAAGCAAAAAGCCCACTGCTCACGCTAGGAACCAAAAGGCCCAGCAGTGATCCAGCCCCAAAAGAGATCTCCTTTGCCCCGGAGATTACAATGGACGATTTCCTCTATCTTTACGATCTAGGAAGGAAGTTCGAAGGTGAAGGTGACGACACTATGTTCACCACTGATAATGAGAAGGTTAGCCTCTTCAATTTCAGAAAGAATGCTGACCCACAGATGGTTAGAGAGGCCTACGCAGCAGGTCTCATCAAGACGATCTACCCGAGTAACAATCTCCAGGAGATCAAATACCTTCCCAAGAAGGTTAAAGATGCAGTCAAAAGATTCAGGACTAATTGCATCAAGAACACAGAGAAAGACATATTTCTCAAGATCAGAAGTACTATTCCAGTATGGACGATTCAAGGCTTGCTTCATAAACCAAGGCAAGTAATAGAGATTGGAGTCTCTAAAAAGGTAGTTCCTACTGAATCTAAGGCCATGCATGGAGTCTAAGATTCAAATCGAGGATCTAACAGAACTCGCCGTGAAGACTGGCGAACAGTTCATACAGAGTCTTTTACGACTCAATGACAAGAAGAAAATCTTCGTCAACATGGTGGAGCACGACACTCTGGTCTACTCCAAAAATGTCAAAGATACAGTCTCAGAAGACCAAAGGGCTATTGAGACTTTTCAACAAAGGATAATTTCGGGAAACCTCCTCGGATTCCATTGCCCAGCTATCTGTCACTTCATCGAAAGGACAGTAGAAAAGGAAGGTGGCTCCTACAAATGCCATCATTGCGATAAAGGAAAGGCTATCATTCAAGATCTGCCTCTGCCGACAGTGGTCCCAAAGATGGACCCCCACCCACGAGGAGCATCGTGGAAAAAGAAGACGTTCCAACCACGTCTTCAAAGCAAGTGGATTGATGTGACATCTCCACTGACGTAAGGGATGACGCACAATCCCACTATCCTTCGCAAGACCCTTCCTCTATATAAGGAAGTTCATTTCATTTGGAGAGGACACGCtcgagatcacaagtttgtacaaaaaagcaggctccgaattcgcccttcaccatggataagaagtactctatcggactcgatatcggaactaactctgtgggatgggctgtgatcaccgatgagtacaaggtgccatctaagaagttcaaggttctcggaaacaccgataggcactctatcaagaaaaaccttatcggtgctctcctcttcgattctggtgaaactgctgaggctaccagactcaagagaaccgctagaagaaggtacaccagaagaaagaacaggatctgctacctccaagagatcttctctaacgagatggctaaagtggatgattcattcttccacaggctcgaagagtcattcctcgtggaagaagataagaagcacgagaggcaccctatcttcggaaacatcgttgatgaggtggcataccacgagaagtaccctactatctaccacctcagaaagaagctcgttgattctactgataaggctgatctcaggctcatctacctcgctctcgctcacatgatcaagttcagaggacacttcctcatcgagggtgatctcaaccctgataactctgatgtggataagttgttcatccagctcgtgcagacctacaaccagcttttcgaagagaaccctatcaacgcttcaggtgtggatgctaaggctatcctctctgctaggctctctaagtcaagaaggcttgagaacctcattgctcagctccctggtgagaagaagaacggacttttcggaaacttgatcgctctctctctcggactcacccctaacttcaagtctaacttcgatctcgctgaggatgcaaagctccagctctcaaaggatacctacgatgatgatctcgataacctcctcgctcagatcggagatcagtacgctgatttgttcctcgctgctaagaacctctctgatgctatcctcctcagtgatatcctcagagtgaacaccgagatcaccaaggctccactctcagcttctatgatcaagagatacgatgagcaccaccaggatctcacacttctcaaggctcttgttagacagcagctcccagagaagtacaaagagattttcttcgatcagtctaagaacggatacgctggttacatcgatggtggtgcatctcaagaagagttctacaagttcatcaagcctatcctcgagaagatggatggaaccgaggaactcctcgtgaagctcaatagagaggatcttctcagaaagcagaggaccttcgataacggatctatccctcatcagatccacctcggagagttgcacgctatccttagaaggcaagaggatttctacccattcctcaaggataacagggaaaagattgagaagattctcaccttcagaatcccttactacgtgggacctctcgctagaggaaactcaagattcgcttggatgaccagaaagtctgaggaaaccatcaccccttggaacttcgaagaggtggtggataagggtgctagtgctcagtctttcatcgagaggatgaccaacttcgataagaaccttccaaacgagaaggtgctccctaagcactctttgctctacgagtacttcaccgtgtacaacgagttgaccaaggttaagtacgtgaccgagggaatgaggaagcctgcttttttgtcaggtgagcaaaagaaggctatcgttgatctcttgttcaagaccaacagaaaggtgaccgtgaagcagctcaaagaggattacttcaagaaaatcgagtgcttcgattcagttgagatttctggtgttgaggataggttcaacgcatctctcggaacctaccacgatctcctcaagatcattaaggataaggatttcttggataacgaggaaaacgaggatatcttggaggatatcgttcttaccctcaccctctttgaagatagagagatgattgaagaaaggctcaagacctacgctcatctcttcgatgataaggtgatgaagcagttgaagagaagaagatacactggttggggaaggctctcaagaaagctcattaacggaatcagggataagcagtctggaaagacaatccttgatttcctcaagtctgatggattcgctaacagaaacttcatgcagctcatccacgatgattctctcacctttaaagaggatatccagaaggctcaggtttcaggacagggtgatagtctccatgagcatatcgctaacctcgctggatctcctgcaatcaagaagggaatcctccagactgtgaaggttgtggatgagttggtgaaggtgatgggaaggcataagcctgagaacatcgtgatcgaaatggctagagagaaccagaccactcagaagggacagaagaactctagggaaaggatgaagaggatcgaggaaggtatcaaagagcttggatctcagatcctcaaagagcaccctgttgagaacactcagctccagaatgagaagctctacctctactacctccagaacggaagggatatgtatgtggatcaagagttggatatcaacaggctctctgattacgatgttgatcatatcgtgccacagtcattcttgaaggatgattctatcgataacaaggtgctcaccaggtctgataagaacaggggtaagagtgataacgtgccaagtgaagaggttgtgaagaaaatgaagaactattggaggcagctcctcaacgctaagctcatcactcagagaaagttcgataacttgactaaggctgagaggggaggactctctgaattggataaggcaggattcatcaagaggcagcttgtggaaaccaggcagatcactaagcacgttgcacagatcctcgattctaggatgaacaccaagtacgatgagaacgataagttgatcagggaagtgaaggttatcaccctcaagtcaaagctcgtgtctgatttcagaaaggatttccaattctacaaggtgagggaaatcaacaactaccaccacgctcacgatgcttaccttaacgctgttgttggaaccgctctcatcaagaagtatcctaagctcgagtcagagttcgtgtacggtgattacaaggtgtacgatgtgaggaagatgatcgctaagtctgagcaagagatcggaaaggctaccgctaagtatttcttctactctaacatcatgaatttcttcaagaccgagattaccctcgctaacggtgagatcagaaagaggccactcatcgagacaaacggtgaaacaggtgagatcgtgtgggataagggaagggatttcgctaccgttagaaaggtgctctctatgccacaggtgaacatcgttaagaaaaccgaggtgcagaccggtggattctctaaagagtctatcctccctaagaggaactctgataagctcattgctaggaagaaggattgggaccctaagaaatacggtggtttcgattctcctaccgtggcttactctgttctcgttgtggctaaggttgagaagggaaagagtaagaagctcaagtctgttaaggaacttctcggaatcactatcatggaaaggtcatctttcgagaagaacccaatcgatttcctcgaggctaagggatacaaagaggttaagaaggatctcatcatcaagctcccaaagtactcactcttcgaactcgagaacggtagaaagaggatgctcgcttctgctggtgagcttcaaaagggaaacgagcttgctctcccatctaagtacgttaactttctttacctcgcttctcactacgagaagttgaagggatctccagaagataacgagcagaagcaacttttcgttgagcagcacaagcactacttggatgagatcatcgagcagatctctgagttctctaaaagggtgatcctcgctgatgcaaacctcgataaggtgttgtctgcttacaacaagcacagagataagcctatcagggaacaggcagagaacatcatccatctcttcacccttaccaacctcggtgctcctgctgctttcaagtacttcgatacaaccatcgataggaagagatacacctctaccaaagaagtgctcgatgctaccctcatccatcagtctatcactggactctacgagactaggatcgatctctcacagctcggtggtgattcaagggctgatcctaagaagaagaggaaggtttgaggcgcgccgagctctctagagctagcgtttaaacaccggtgacgtccgatcgttcaaacatttggcaataaagtttcttaagattgaatcctgttgccggtcttgcgatgattatcatataatttctgttgaattacgttaagcatgtaataattaacatgtaatgcatgacgttatttatgagatgggtttttatgattagagtcccgcaattatacatttaatacgcgatagaaaacaaaatatagcgcgcaaactaggataaattatcgcgcgcggtgtcatctatgttactagatcgggaattgatcccccctcgacagcttccggaaagggcgaattcgcaactttgtatacaaaagttgccccatggcgttccctctagataacgcaggatccccaagtggtggctattttactttaaatttttcttatggctcagcctgtgatggataactgaatcaaacaaatggcgtctgggtttaagaacatctgttttggctatgttggacgaaacaagtgaacttttaggatcaacttccgtttatatacggagcttatatcgagcaataagataagtgggctttttatgtaatttaatgggctatcgtccatatattcactaatacccatgcccagtacccatgtatgcgtttcatataagctcctaatttctcccacatcgctcaaatctaaacaaatcttgttgtatatataacactgagggagcaccattggtcaGGTCGTTGGAGGTAGTGTGGgttttagagctagaaatagcaagttaaaataaggctagtccgttatcaacttgaaaaagtggcaccgagtcggtgcttttttttccctttccttttttcttttttttgccataaacttaaatttgtatatcgatcattgtagatattgaaaacctagaacaaaccaacatccatgtgaatgtctttcatgactgatttagagataattcttgaattttggaactagaatctataatgagcctaaattaaaacattgtgcatgagaaatctcaaaattccggcagaacaattttgaatctcgatccgtagaaaccagacggtcattgttttagttccaccacgattatatttgaaatttacgtgagtgtgagtgagacttgcataagaaaataaaatctttagttgggaaaaaattcaataatataaatgggcttgagaaggaagcgagggataggcctttttctaaaataggcccatttaagctattaacaatcttcaaaagtaccacagcgcttaggtaaagaaagcagctgagtttatatatggttagacacgaagtagtgattGGACAAGGGCAGGTCGTTGGgttttagagctagaaatagcaagttaaaataaggctagtccgttatcaacttgaaaaagtggcaccgagtcggtgctttttttggcaaaaattttcagattttttcttcatctgtagatttctgggtttttttttccgtttcgtgaatcataagtgaagttttggatgcaaatctgcgcgaaaaaagttggacctgcaatgagcttatttagatagctaagacaaagtgattggtccgttgtttcagttctgattgtcagagagtttgtttcgagtcggcgacaccaatgcgttttgttaaccagatttcgggtaagaaatgtatcgagagtttgtttcaagacggctacatcattttcttatgaagggtgaaattagatagaccaaagattgaaacacaacatttctttcacaaaaatataataaacttgatagcatttaggatcagcGGACactagtaagggcgaattcgacccagctttcttgtacaaagtggtgCCTAGGTGAGTCTAGAGAGTTAATTAAGACCCGGGACTAgtccctagagtcctGTCTTTAATGAGATATGCGAGACGCCTATGATCGCATGATATTTGCTTTCAATTCTGTTGTGCACGTTGTAAAAAACCTGAGCATGTGTAGCTCAGATCCTTACCGCCGGTTTCGGTTCATTCTAATGAATATATCACCCGTTACTATCGTATTTTTATGAATAATATTCTCCGTTCAATTTaCTGATTGTACCCTACTACTTATATGTACAATATTAAAATGAAAACAATATATTGTGCTGAATAGGTTTATAGCGACATCTATGATAGAGCGCCACAATAACAAACAATTGCGTTTTATTATTACAAATCCAATTTTAAAAAAAGCGGCAGAACCGGTCAAACCTAAAAGACTGATTACATAAATCTTATTCAAATTTCAAAAGTGCCCCAGGGGCTAGTATCTACGACACACCGAGCGGCGAACTAATAACGCTCACTGAAGGGAACTCCGGTTCcCCGCCGGCGCGCATGGGTGAGATTCCTTGAAGTTGAGTATTGGCCGTCCGCTCTACCGAAAGTTACGGGCACCATTCAACCCGGTCCAGCACGGCGGCCGGGTAACCGACTTGCTGCCCCGAGAATTATGCAGCATTTTTTTGGTGTATGTGGGCCCCAAATGAAGTGCAGGTCAAACCTTGACAGTGACGACAAATCGTTGGGCGGGTCCAGGGCGAATTTTGCGACAACATGTCGAGGCTCAGCAGgacCTGCAGGCATGCAagcttggcactggccgtcgttttacaacgtcgtgactgggaaaaccctggcgttacccaacttaatcgccttgcagcacatccccctttcgccagctggcgtaatagcgaagaggcccgcaccgatcgcccttcccaacagttgcgcagcctgaatggcgaatgctagagcagcttgagcttggatcagattgtcgtttcccgccttcagtttaaactatcagtgtttgacaggatatattggcgggtaaacctaagagaaaagagcgtttattagaataacggatatttaaaagggcgtgaaaaggtttatccgttcgtccatttgtatgtgcatgccaaccacagggttcccctcgggatcaaagtactttgatccaacccctccgctgctatagtgcagtcggcttctgacgttcagtgcagccgtcttctgaaaacgacatgtcgcacaagtcctaagttacgcgacaggctgccgccctgcccttttcctggcgttttcttgtcgcgtgttttagtcgcataaagtagaatacttgcgactagaaccggagacattacgccatgaacaagagcgccgccgctggcctgctgggctatgcccgcgtcagcaccgacgaccaggacttgaccaaccaacgggccgaactgcacgcggccggctgcaccaagctgttttccgagaagatcaccggcaccaggcgcgaccgcccggagctggccaggatgcttgaccacctacgccctggcgacgttgtgacagtgaccaggctagaccgcctggcccgcagcacccgcgacctactggacattgccgagcgcatccaggaggccggcgcgggcctgcgtagcctggcagagccgtgggccgacaccaccacgccggccggccgcatggtgttgaccgtgttcgccggcattgccgagttcgagcgttccctaatcatcgaccgcacccggagcgggcgcgaggccgccaaggcccgaggcgtgaagtttggcccccgccctaccctcaccccggcacagatcgcgcacgcccgcgagctgatcgaccaggaaggccgcaccgtgaaagaggcggctgcactgcttggcgtgcatcgctcgaccctgtaccgcgcacttgagcgcagcgaggaagtgacgcccaccgaggccaggcggcgcggtgccttccgtgaggacgcattgaccgaggccgacgccctggcggccgccgagaatgaacgccaagaggaacaagcatgaaaccgcaccaggacggccaggacgaaccgtttttcattaccgaagagatcgaggcggagatgatcgcggccgggtacgtgttcgagccgcccgcgcacgtctcaaccgtgcggctgcatgaaatcctggccggtttgtctgatgccaagctggcggcctggccggccagcttggccgctgaagaaaccgagcgccgccgtctaaaaaggtgatgtgtatttgagtaaaacagcttgcgtcatgcggtcgctgcgtatatgatgcgatgagtaaataaacaaatacgcaaggggaacgcatgaaggttatcgctgtacttaaccagaaaggcgggtcaggcaagacgaccatcgcaacccatctagcccgcgccctgcaactcgccggggccgatgttctgttagtcgattccgatccccagggcagtgcccgcgattgggcggccgtgcgggaagatcaaccgctaaccgttgtcggcatcgaccgcccgacgattgaccgcgacgtgaaggccatcggccggcgcgacttcgtagtgatcgacggagcgccccaggcggcggacttggctgtgtccgcgatcaaggcagccgacttcgtgctgattccggtgcagccaagcccttacgacatatgggccaccgccgacctggtggagctggttaagcagcgcattgaggtcacggatggaaggctacaagcggcctttgtcgtgtcgcgggcgatcaaaggcacgcgcatcggcggtgaggttgccgaggcgctggccgggtacgagctgcccattcttgagtcccgtatcacgcagcgcgtgagctacccaggcactgccgccgccggcacaaccgttcttgaatcagaacccgagggcgacgctgcccgcgaggtccaggcgctggccgctgaaattaaatcaaaactcatttgagttaatgaggtaaagagaaaatgagcaaaagcacaaacacgctaagtgccggccgtccgagcgcacgcagcagcaaggctgcaacgttggccagcctggcagacacgccagccatgaagcgggtcaactttcagttgccggcggaggatcacaccaagctgaagatgtacgcggtacgccaaggcaagaccattaccgagctgctatctgaatacatcgcgcagctaccagagtaaatgagcaaatgaataaatgagtagatgaattttagcggctaaaggaggcggcatggaaaatcaagaacaaccaggcaccgacgccgtggaatgccccatgtgtggaggaacgggcggttggccaggcgtaagcggctgggttgtctgccggccctgcaatggcactggaacccccaagcccgaggaatcggcgtgacggtcgcaaaccatccggcccggtacaaatcggcgcggcgctgggtgatgacctggtggagaagttgaaggccgcgcaggccgcccagcggcaacgcatcgaggcagaagcacgccccggtgaatcgtggcaagcggccgctgatcgaatccgcaaagaatcccggcaaccgccggcagccggtgcgccgtcgattaggaagccgcccaagggcgacgagcaaccagattttttcgttccgatgctctatgacgtgggcacccgcgatagtcgcagcatcatggacgtggccgttttccgtctgtcgaagcgtgaccgacgagctggcgaggtgatccgctacgagcttccagacgggcacgtagaggtttccgcagggccggccggcatggccagtgtgtgggattacgacctggtactgatggcggtttcccatctaaccgaatccatgaaccgataccgggaagggaagggagacaagcccggccgcgtgttccgtccacacgttgcggacgtactcaagttctgccggcgagccgatggcggaaagcagaaagacgacctggtagaaacctgcattcggttaaacaccacgcacgttgccatgcagcgtacgaagaaggccaagaacggccgcctggtgacggtatccgagggtgaagccttgattagccgctacaagatcgtaaagagcgaaaccgggcggccggagtacatcgagatcgagctagctgattggatgtaccgcgagatcacagaaggcaagaacccggacgtgctgacggttcaccccgattactttttgatcgatcccggcatcggccgttttctctaccgcctggcacgccgcgccgcaggcaaggcagaagccagatggttgttcaagacgatctacgaacgcagtggcagcgccggagagttcaagaagttctgtttcaccgtgcgcaagctgatcgggtcaaatgacctgccggagtacgatttgaaggaggaggcggggcaggctggcccgatcctagtcatgcgctaccgcaacctgatcgagggcgaagcatccgccggttcctaatgtacggagcagatgctagggcaaattgccctagcaggggaaaaaggtcgaaaaggtctctttcctgtggatagcacgtacattgggaacccaaagccgtacattgggaaccggaacccgtacattgggaacccaaagccgtacattgggaaccggtcacacatgtaagtgactgatataaaagagaaaaaaggcgatttttccgcctaaaactctttaaaacttattaaaactcttaaaacccgcctggcctgtgcataactgtctggccagcgcacagccgaagagctgcaaaaagcgcctacccttcggtcgctgcgctccctacgccccgccgcttcgcgtcggcctatcgcggccgctggccgctcaaaaatggctggcctacggccaggcaatctaccagggcgcggacaagccgcgccgtcgccactcgaccgccggcgcccacatcaaggcaccctgcctcgcgcgtttcggtgatgacggtgaaaacctctgacacatgcagctcccggagacggtcacagcttgtctgtaagcggatgccgggagcagacaagcccgtcagggcgcgtcagcgggtgttggcgggtgtcggggcgcagccatgacccagtcacgtagcgatagcggagtgtatactggcttaactatgcggcatcagagcagattgtactgagagtgcaccatatgcggtgtgaaataccgcacagatgcgtaaggagaaaataccgcatcaggcgctcttccgcttcctcgctcactgactcgctgcgctcggtcgttcggctgcggcgagcggtatcagctcactcaaaggcggtaatacggttatccacagaatcaggggataacgcaggaaagaacatgtgagcaaaaggccagcaaaaggccaggaaccgtaaaaaggccgcgttgctggcgtttttccataggctccgcccccctgacgagcatcacaaaaatcgacgctcaagtcagaggtggcgaaacccgacaggactataaagataccaggcgtttccccctggaagctccctcgtgcgctctcctgttccgaccctgccgcttaccggatacctgtccgcctttctcccttcgggaagcgtggcgctttctcatagctcacgctgtaggtatctcagttcggtgtaggtcgttcgctccaagctgggctgtgtgcacgaaccccccgttcagcccgaccgctgcgccttatccggtaactatcgtcttgagtccaacccggtaagacacgacttatcgccactggcagcagccactggtaacaggattagcagagcgaggtatgtaggcggtgctacagagttcttgaagtggtggcctaactacggctacactagaaggacagtatttggtatctgcgctctgctgaagccagttaccttcggaaaaagagttggtagctcttgatccggcaaacaaaccaccgctggtagcggtggtttttttgtttgcaagcagcagattacgcgcagaaaaaaaggatctcaagaagatcctttgatcttttctacggggtctgacgctcagtggaacgaaaactcacgttaagggattttggtcatgcattctaggtactaCCAATGCTTAATCAGTGAGGCACCTATCTCAGCGATCTGTCTATTTCGTTCATCCATAGTTGCCTGACTCCCCGTCGTGTAGATAACTACGATACGGGAGGGCTTACCATCTGGCCCCAGTGCTGCAATGATACCGCGAGACCCACGCTCACCGGCTCCAGATTTATCAGCAATAAACCAGCCAGCCGGAAGGGCCGAGCGCAGAAGTGGTCCTGCAACTTTATCCGCCTCCATCCAGTCTATTAATTGTTGCCGGGAAGCTAGAGTAAGTAGTTCGCCAGTTAATAGTTTGCGCAACGTTGTTGCCATTGCTACAGGCATCGTGGTGTCACGCTCGTCGTTTGGTATGGCTTCATTCAGCTCCGGTTCCCAACGATCAAGGCGAGTTACATGATCCCCCATGTTGTGCAAAAAAGCGGTTAGCTCCTTCGGTCCTCCGATCGTTGTCAGAAGTAAGTTGGCCGCAGTGTTATCACTCATGGTTATGGCAGCACTGCATAATTCTCTTACTGTCATGCCATCCGTAAGATGCTTTTCTGTGACTGGTGAGTACTCAACCAAGTCATTCTGAGAATAGTGTATGCGGCGACCGAGTTGCTCTTGCCCGGCGTCAATACGGGATAATACCGCGCCACATAGCAGAACTTTAAAAGTGCTCATCATTGGAAAACGTTCTTCGGGGCGAAAACTCTCAAGGATCTTACCGCTGTTGAGATCCAGTTCGATGTAACCCACTCGTGCACCCAACTGATCTTCAGCATCTTTTACTTTCACCAGCGTTTCTGGGTGAGCAAAAACAGGAAGGCAAAATGCCGCAAAAAAGGGAATAAGGGCGACACGGAA

**S2: Complete vector sequence**

S3


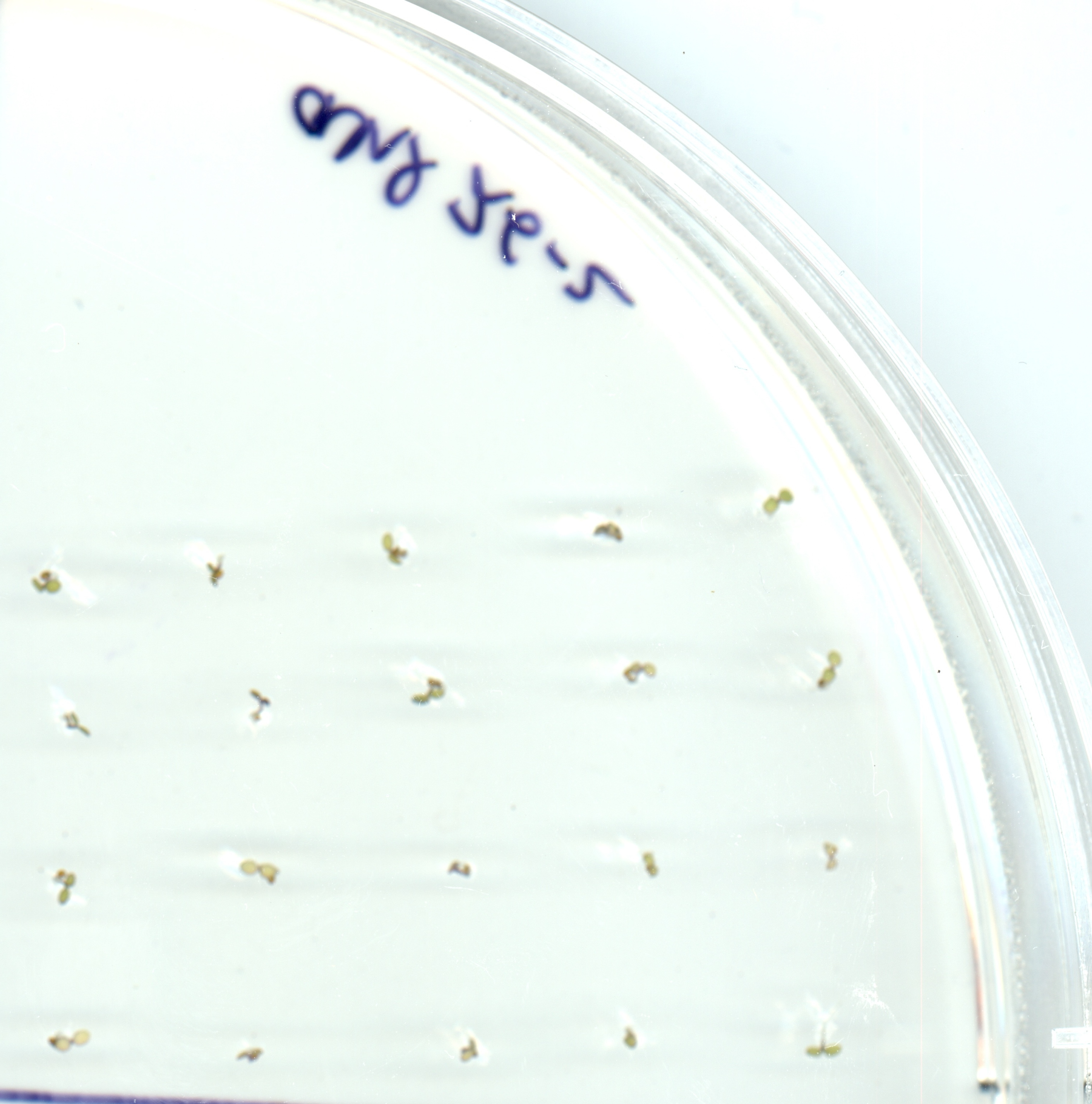


**S3: Necrotic-looking seedlings on Basta media.**


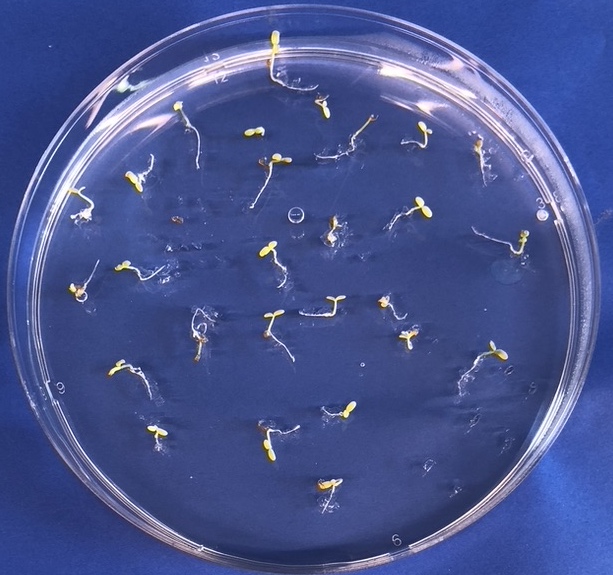

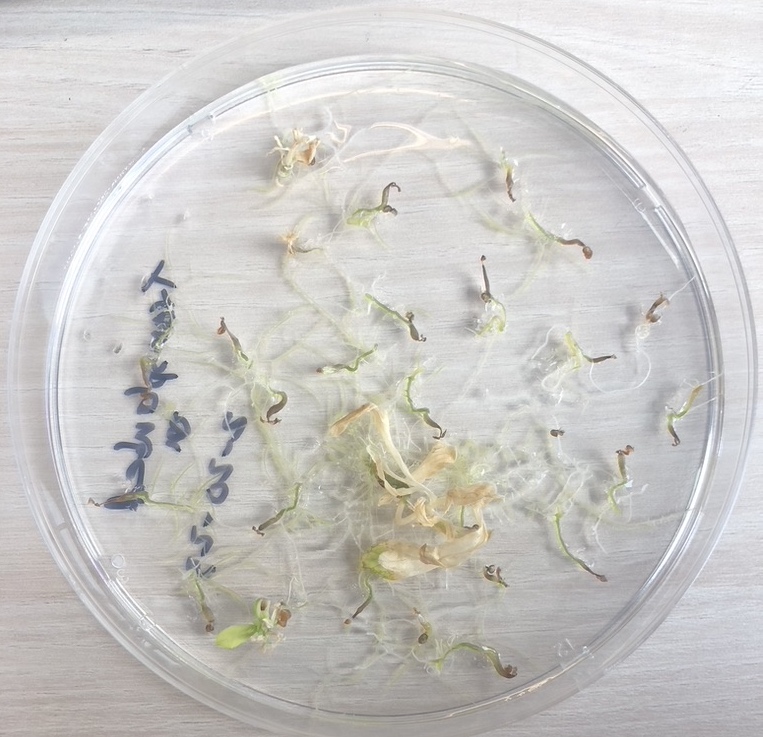


**S4: Camelina recovery on media. Camelina growing on 25 mg/L Basta (left), recovery of camelina after transplanting from Basta media to normal growth media (right)**


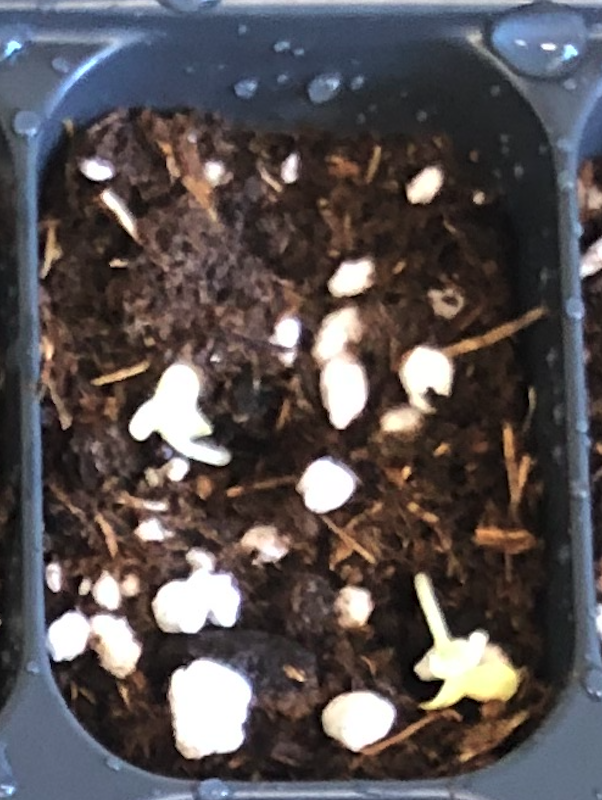

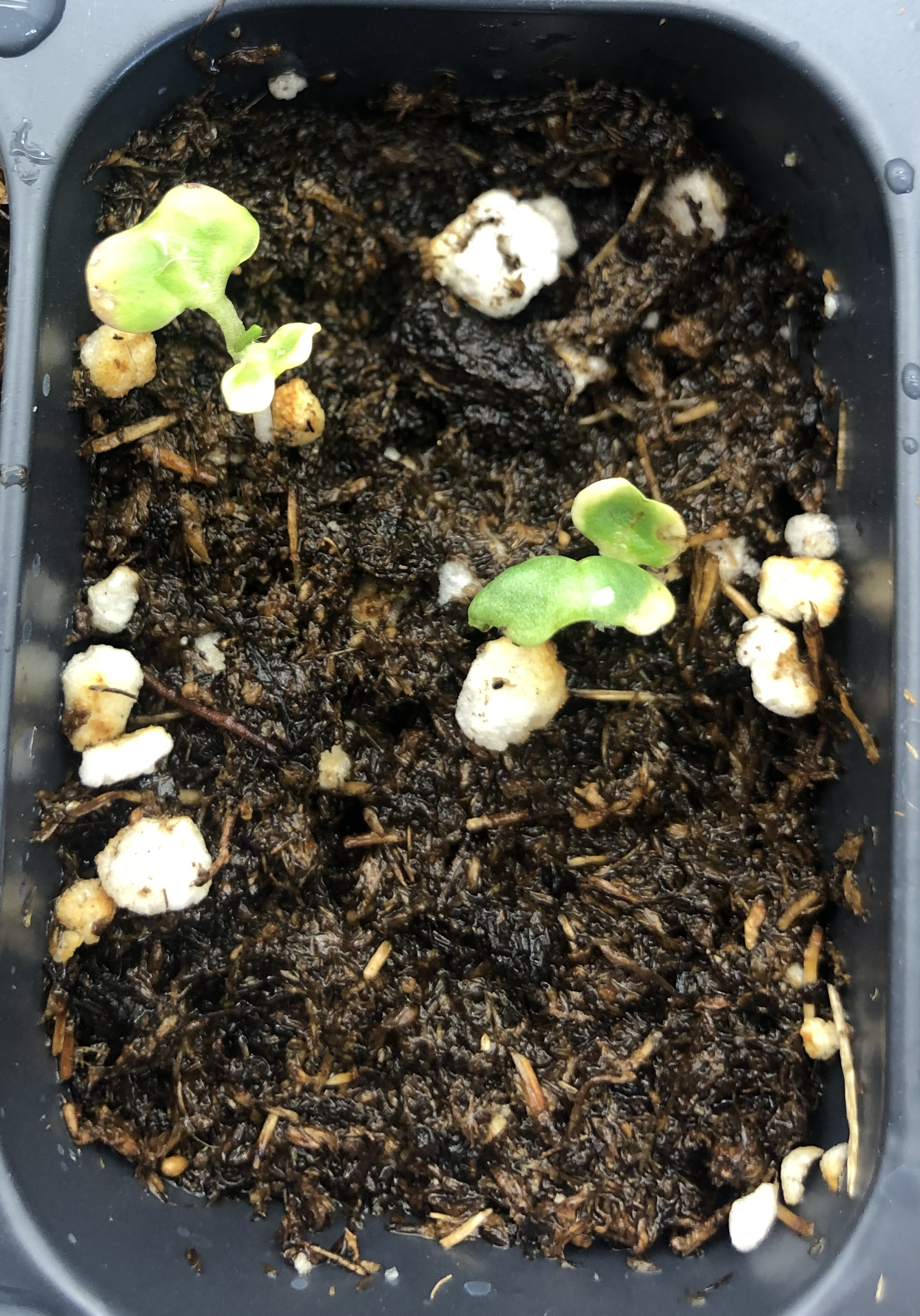


**S5: Canola recovery in soil. Canola transplanted directly to soil from media containing 25mg/L Basta (left), recovery of canola in soil (right)**


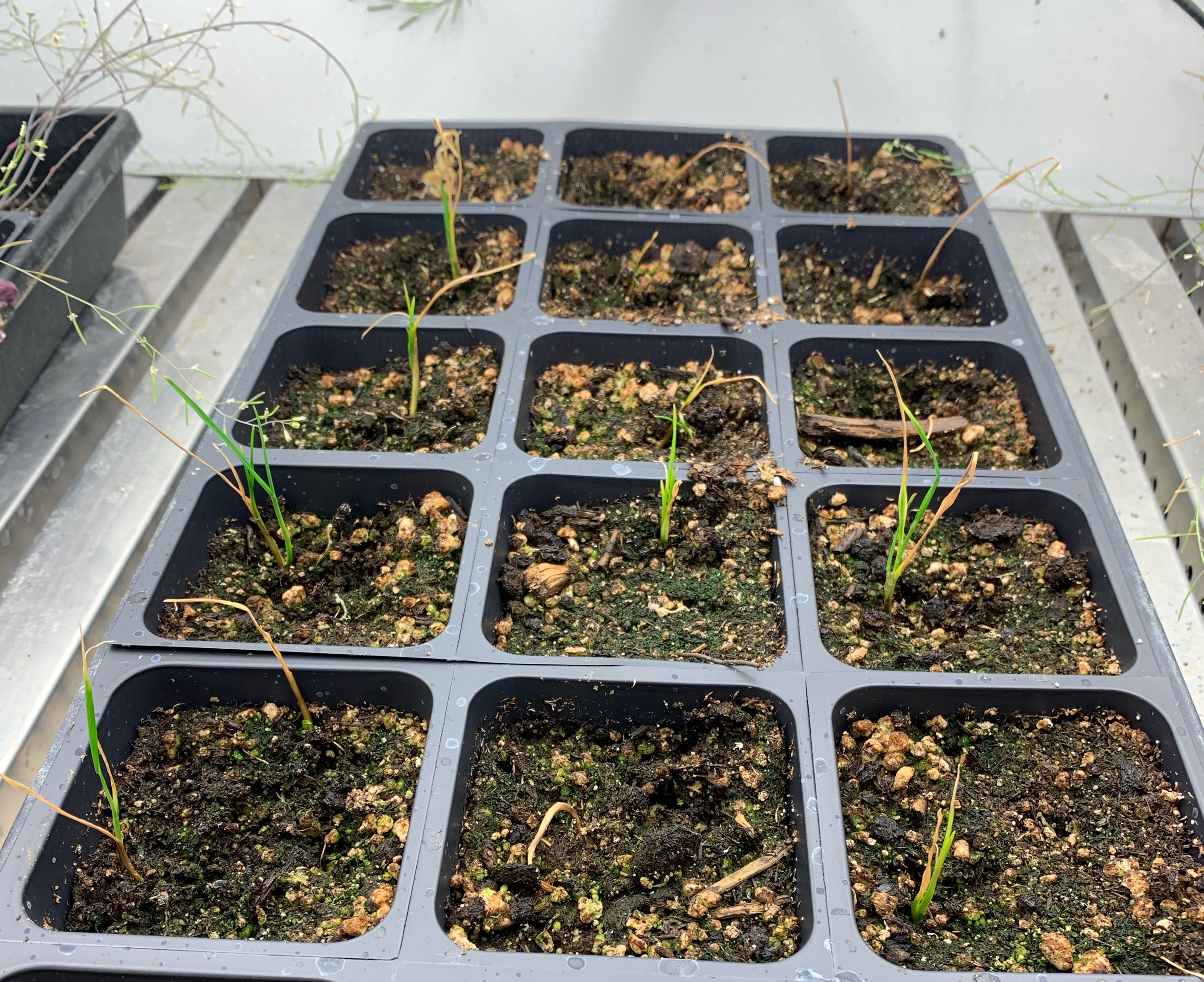


**S6: Wheat recovery in soil after transplanted directly to soil from media containing 25mg/L Basta**
